# Dynamic exchange of two types of stator units in *Bacillus subtilis* flagellar motor in response to environmental changes

**DOI:** 10.1016/j.csbj.2020.10.009

**Published:** 2020-10-15

**Authors:** Naoya Terahara, Keiichi Namba, Tohru Minamino

**Affiliations:** a Department of Physics, Faculty of Science and Engineering, Chuo University, 1-13-27 Kasuga, Bunkyo-ku, Tokyo 112-8551, Japan; b Graduate School of Frontier Bioscience, Osaka University, 1-3 Yamadaoka, Suita, Osaka 565-0871, Japan; c RIKEN Spring-8 Center and Center for Biosystems Dynamics Research, 1-3 Yamadaoka, Suita, Osaka 565-0871, Japan; d JEOL YOKOGUSHI Research Alliance Laboratories, Osaka University, 1-3 Yamadaoka, Suita, Osaka 565-0871, Japan

**Keywords:** *Bacillus subtilis*, Bacterial flagella, Biosensor, Ion channel, Motility, Torque generation

## Abstract

Bacteria can migrate towards more suitable environments by rotating flagella that are under the control of sensory signal transduction networks. The bacterial flagellum is composed of the long helical filament functioning as a propeller, the flexible hook as a universal joint and the basal body as a rotary motor powered by ion motive force across the cell membrane. The flagellar motor consists of a rotor and multiple stator units, each of which couples the ion flow through its ion channel with force generation. The flagellar building blocks and motor proteins are highly conserved among bacterial species, but structural and functional diversity of flagella has also been revealed. It has been reported that the structure and function of the flagellar motor of a Gram-positive bacterium, *Bacillus subtilis*, differ from those of *Escherichia coli* and *Salmonella*. The flagellar motor of the *B. subtilis* BR151MA strain possesses two distinct types of stator complexes, H^+^-type MotAB and Na^+^-type MotPS, around the rotor. These two types of stator units dynamically assemble to and disassemble from the rotor in response to environmental changes such as viscosity and external Na^+^ concentrations. In this mini-review article, we describe our recent understanding of the structure and dynamics of the *B. subtilis* flagellar motor.

## 1 Introduction

**Fig. 1:**
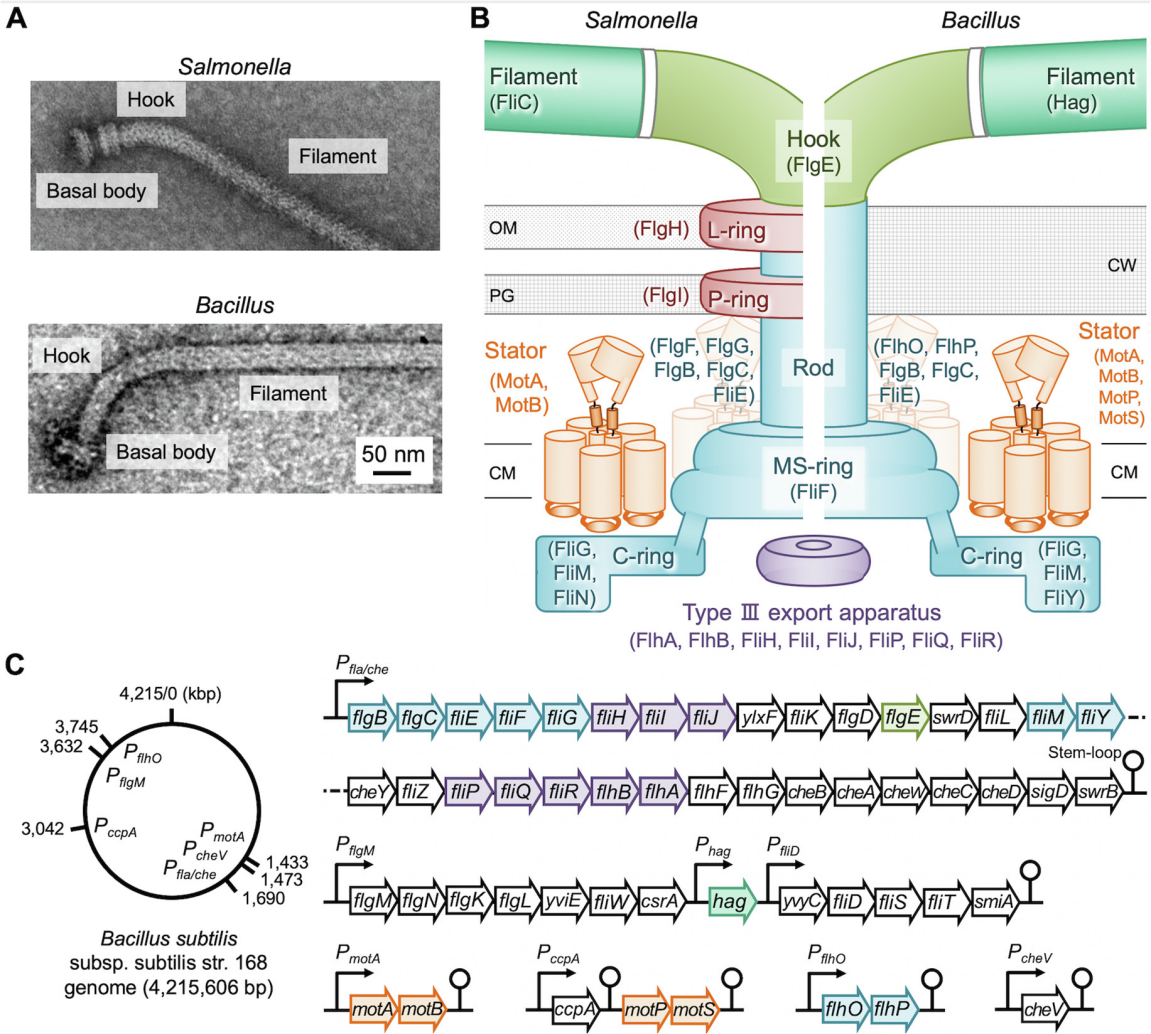
Bacterial flagella. (A) Electron micrographs of purified flagella isolated from *Salmonella* (upper panel) and *B. subtilis* (lower panel). (B) Schematic diagrams of the *Salmonella* and *B. subtilis* flagella. The flagellum is divided into three structural parts: the helical filament that acts as a propeller, the basal body embedded in the cytoplasmic membrane, and the hook that connects the filament to the basal body. The basal body serves as a bi-directional rotary motor composed of a rotor ring complex and multiple stator units around the rotor. CM, cytoplasmic membrane; PG, peptidoglycan layer; OM, outer membrane; CW, cell wall. (C) Genome map and flagellar gene clusters of *B. subtilis*. Different colors mean different structural components of the flagellum. The filament is colored in forest green, the hook is in green, the L P ring complex is in red, the core structure of the basal body (MS ring, C ring, rod) is in cyan, the stator unit is in orange, the flagellar protein export apparatus is in purple. White color represents gene regulators, flagellar export chaperones and chemotaxis proteins. (For interpretation of the references to color in this figure legend, the reader is referred to the web version of this article.)

Many motile bacteria can swim in liquids by rotating their filamentous organelles called flagella and migrate to places of more favorable conditions by sensing external stimuli via sensory signal transducers. The bacterial flagellum is a rotary nanomachine composed of more than 25 different proteins and consists of at least three structural parts: the long helical filament acting as a propeller to produce thrust, the basal body as a membrane-embedded rotary motor, and the hook that acts as a universal joint connecting the filament to the basal body (Fig. 1A, B). The structure, function and assembly of the flagellum are best understood with *Escherichia coli* and *Salmonella enterica* serovar Typhimurium (hereafter referred to *Salmonella*), which are both Gram-negative bacteria. The flagellar motor of *E. coli* and *Salmonella* utilizes the electrochemical potential of protons across the cytoplasmic membrane, namely proton motive force (PMF), as the energy source to rotate the flagellar filament [1], [2], [3], [4], [5], [6].

The flagellar motor of *E. coli* and *Salmonella* consists of a rotor ring complex and multiple stator units around the rotor (Fig. 1B). The rotor ring complex is composed of the MS ring made of a transmembrane protein, FliF, and the C ring consisting of three cytoplasmic proteins, FliG, FliM and FliN. The C ring serves as the switching device responsible for the control of the direction of flagellar rotation [counterclockwise (CCW) or clockwise (CW)]. This switching device is placed under the control of the chemotaxis signaling networks, where the signaling molecule CheY-phosphate (CheY-P) binds to the switching device to induce a switch from CCW to CW rotation. As a result, *E. coli* and *Salmonella* cells stop swimming and change the swimming direction to move toward more favorable environments [7]. Two transmembrane proteins, MotA and MotB, form a proton (H^+^) channel complex that acts as a stator unit to couple the H^+^ flow through the channel with force generation for high-speed rotation of the filament [8].

A Gram-positive bacterium, *Bacillus subtilis*, has multiple flagella on the cell surface in a way similar to *E. coli* and *Salmonella*, but the structure and function of the *B. subtilis* flagella are different from those of *E. coli* and *Salmonella*. For example, CheY-P binds to the switching device of the *B. subtilis* flagellar motor to induce a switch from CW to CCW rotation, opposite to the action in *E. coli* and *Salmonella* [9]. The *B. subtilis* flagellar motor activity is also controlled by two speed modulation proteins, namely EpsE and MotI, both of which inhibit flagella-driven motility, thereby efficiently inducing a motility-to-biofilm transition when *B. subtilis* cells attach to solid surfaces [10].

*B. subtilis* BR151MA, which is a derivative of the *B. subtilis* laboratory strain 168, is used as a wild-type strain for motility and chemotaxis studies [11]. This BR151MA strain possesses two distinct stator genes, H^+^-type *motAB* and sodium ion (Na^+^)-type *motPS*, at distinct loci from the major *fla/che* region on the genome (Fig. 1C) [11]. The *motA* and *motB* genes form the *motAB* operon, which is placed in the *B. subtilis* flagellar regulon (Fig. 1C). In contrast, the *motP* and *motS* genes form an operon along with the *ccpA* gene, which encodes a central regulator of carbon metabolism, and so are transcribed from the P*_ccpA_* promoter (Fig. 1C) [11]. Because these is no evidence that the expression of MotP and MotS is placed under control of the flagellar gene regulators, this suggests that these two genes do not belong to the flagellar regulon. A stem-loop structure between the *ccpA* and *motP* genes serves as a transcriptional terminator of the *ccpA* gene (Fig. 1C), and hence the transcription levels of the *motP* and *motS* genes are relatively low in the BR151MA strain, although these two distinct stator units are simultaneously expressed [12]. Thus, the BR151MA flagellar motor becomes a hybrid engine consisting of both H^+^-type MotAB and Na^+^-type MotPS stator units around the same rotor ring complex when external Na^+^ concentration and fluid viscosity are high enough [13], [14]. Interestingly, this hybrid motor autonomously changes the ratio of these two distinct stator units incorporated around the rotor in response to environmental changes, such as fluid viscosity and external Na^+^ concentration [13], [14]. This mini-review article covers current understanding of how such stator exchanges occur in the *B. subtilis* BR151MA strain.

## 2 Overall structure of the *B. subtilis* flagellum

The flagellar basal body has been isolated from *B. subtilis* without the C ring attached (Fig. 1A) [15]. The purified *B. subtilis* basal body is composed of the MS ring (FliF) and the rod (FliE, FlgB, FlgC, FllhO, FlhP) (Fig. 1B) but does not contain the LP ring complex seen in *E. coli* and *Salmonella*, which acts as a molecular bushing to support smooth rotation of the rod acting as a drive shaft [16]. Because the peptidoglycan (PG) layers of Gram-positive bacteria are much thicker than those of Gram-negative bacteria, such thick PG layers appear to be able to support smooth high-speed rotation of the rod of the *B. subtilis* flagellar motor without the LP ring as a bushing.

**Fig. 2:**
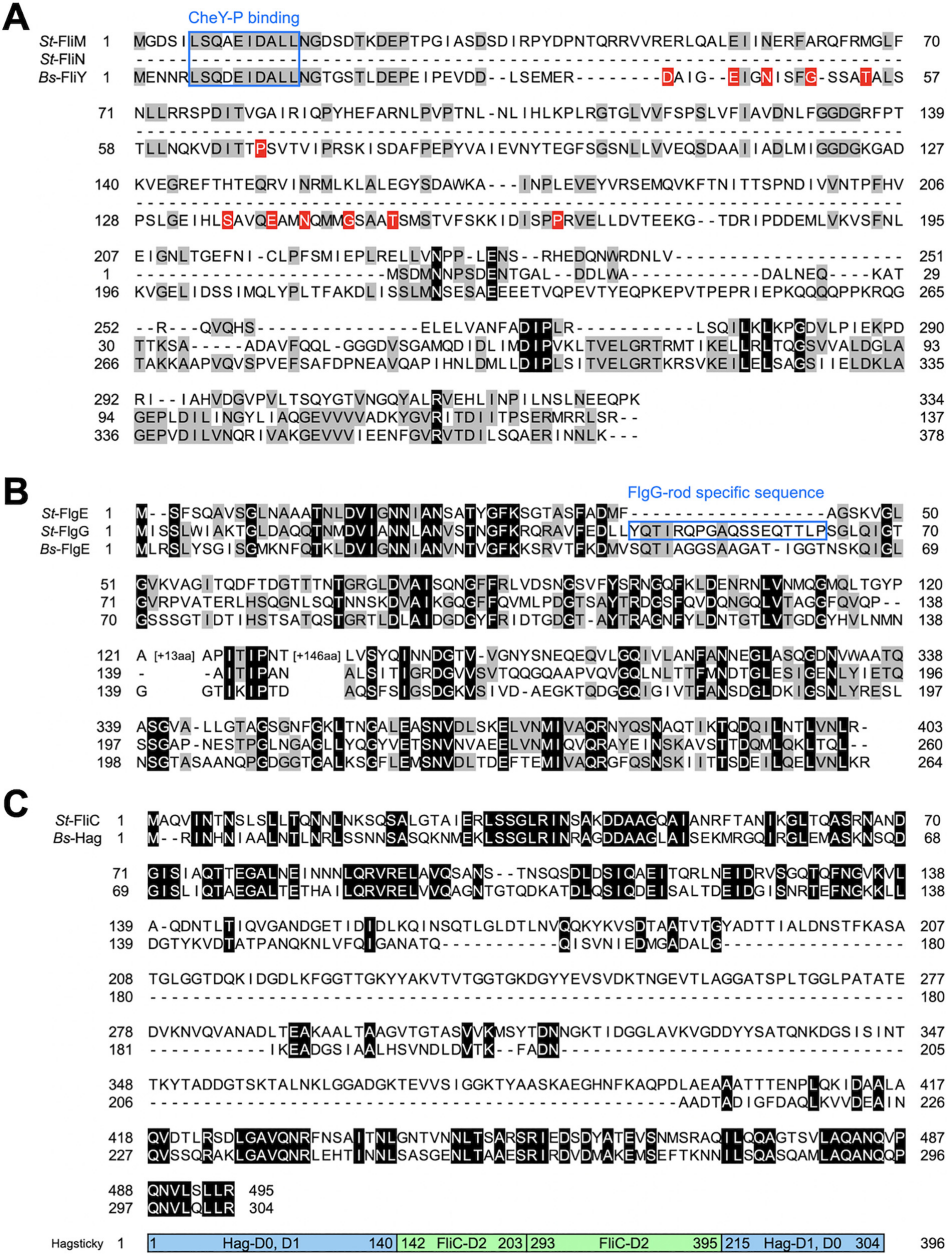
Comparison of flagellar building blocks between *Salmonella* and *B. subtilis* (A) Multiple sequence alignment of *St*-FliM (UniProt ID: P26418), *St*-FliN (P26419) and *Bs*-FliY (P24073). Highly and relative well conserved residues are shown by black and gray boxes, respectively. Red boxes represent amino acid residues responsible for the phosphatase activity. A highly conserved CheY-P binding site is boxed. (B) Multiple sequence alignment of *St*-FlgE (UniProt ID: P0A1J1), *St*-FlgG (P0A1J3) and *Bs*-FlgE (P23446). Highly and relative well conserved residues are shown by black and gray boxes, respectively. The FlgG-rod specific sequence is boxed. (C) Multiple sequence alignment of *St*-FliC (UniProt ID: P06179) and *Bs*-Hag (P02968). Conserved residues are shown by black boxes. FliC and Hag region in the Hag_sticky_ sequence are indicated by color bars, respectively. (For interpretation of the references to color in this figure legend, the reader is referred to the web version of this article.)

**Fig. 3:**
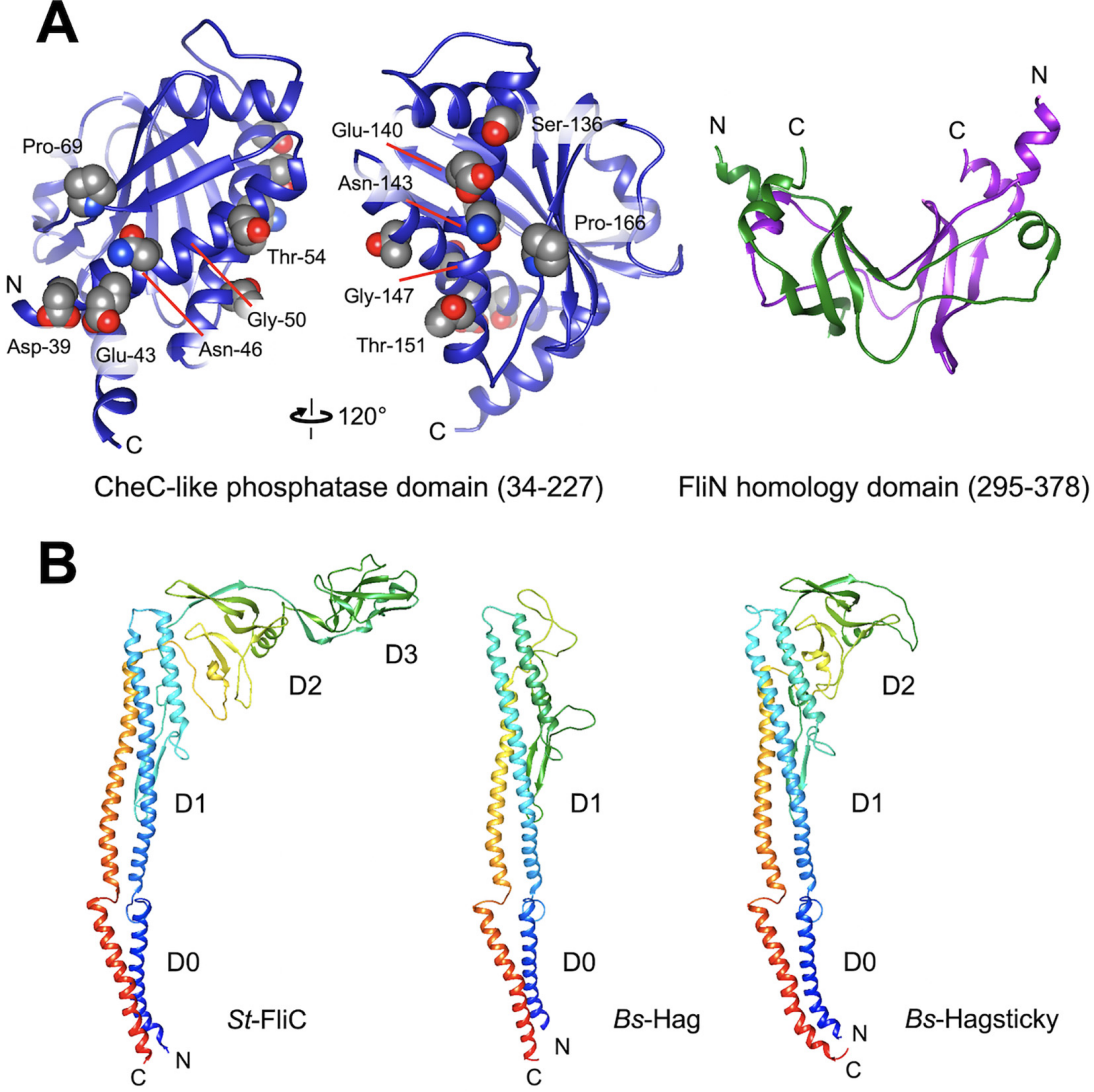
Structural models of FliY and Hag. (A) Homology model of *Bs*-FliY CheC-like phosphatase domain (residues 34–227) and FliN-like domain (residues 295–378) built from the crystal structures of *Thermotoga maritima* FliY and FliN (PDB ID: 4hyn and 1yab). The FliN-homologous domain is shown as a dimer. (B) Homology model of *Bs*-Hag and *Bs*-Hag_sticky_ built from the crystal structure of *St*-FliC (PDB ID: 3a5x).

The *B. subtilis* C ring is composed of FliG, FliM and FliY [17]. *Bs*-FliG and *Bs-*FliM are homologous to the *E. coli* and *Salmonella* counterparts, but *Bs*-FliY shows high sequence similarities to both *St-*FliM and *St-*FliN (Fig. 2A). Like the N-terminal domain of *St-*FliM (*St-*FliM_N_), the N-terminal domain of FliY (*Bs-*FliY_N_) contains a well conserved LSQXEIDALL sequence (Fig. 2A), which is responsible for the interaction with CheY-P [18]. In fact, CheY-P binds to both *Bs-*FliM_N_ and *Bs-*FliY_N_ [19]. The middle domain of FliY (*Bs-*FliY_M_) serves as a phosphatase to promote dephosphorylation of CheY-P (Fig. 3A), thereby inducing the dissociation of CheY to allow the motor to switch its rotational direction from CCW to CW [20]. The C-terminal domain of FliY resembles *St-*FliN and the C-terminal domain of *St-*FliM (Figs. 2A and 3B), thereby allowing *Bs-*FliM and *Bs-*FliY to form the continuous C ring wall on the *Bs-*FliG ring structure in a way similar to the *E. coli* and *Salmonella* C ring structures [17].

The rod is composed of five proteins, FliE, FlgB, FlgC, FlhO and FlhP (Fig. 1C). Unlike *Salmonella*, the *flhO* and *flhP* genes are located at different loci in the genome (Fig. 1D), but it has recently been demonstrated that these gene products assemble into the rod structure [21]. The assembly of the rod begins with FliE, which severs as an adaptor to connect the rod to the polypeptide channel complex of the flagellar protein export apparatus [22], [23], followed by the assembly of FlgB, FlgC, FlhO and FlhP in this order [21].

Since *B. subtilis* FlgE (*Bs*-FlgE) shows an extensive sequence similarity to *Salmonella* distal rod protein FlgG (*St-*FlgG) (Fig. 2B), *Bs*-FlgE was originally thought to be a rod protein. But, it has been demonstrated that *Bs*-FlgE assembles into the hook structure [24]. *Bs*-FlgE consists of three domains, D0, Dc and D1, in a way similar to *St-*FlgG [25]. The Dc domain of *St-*FlgG contains the FlgG-rod specific sequence (GSS; YQTIRQPGAQSSEQTTLP), which stabilizes the straight and rigid FlgG rod structure of *Salmonella*, whereas that of *St-*FlgE does not [26]. Since the insertion of the GSS into the Dc domain of *St-*FlgE actually makes the *Salmonella* hook straight and rigid, the GSS is responsible for the rigidity of the FlgG rod structure [27]. Because *Bs-*FlgE has the GSS-like sequence in the Dc domain albeit one residue shorter than GSS (Fig. 2B) [25], it remains obscure how the *B. subtilis* hook adopts a curved form with a bending flexibility. To clarify this, high-resolution structural analysis will be required.

A single flagellin protein (Hag) assembles into the filament in *B. subtilis* in a way similar to the *Salmonella* filament. Hag consists of only two domains D0 and D1 [28] whereas *Salmonella* flagellin (FliC or FljB) is composed of four domains, D0, D1, D2 and D3 (Fig. 2C and 3B) [29], [30], and so the diameter of the *B. subtilis* filament (~125 Å) is much smaller than that of the *Salmonella* filament (~230 Å). The *B. subtilis* filament undergoes polymorphic transformations of its supercoiled form during motor rotation in a way similar to the *Salmonella* filament [28].

For construction of the flagella on the cell surface, a specialized protein export apparatus transports flagellar building blocks from the cytoplasm to the distal end. The flagellar protein export apparatus is composed of a transmembrane export gate complex made of five membrane proteins, FlhA, FlhB, FliP, FliQ and FliR, and a cytoplasmic ATPase ring complex consisting of FliH, FliI and FliJ (Fig. 1C) [31], [32]. The structure and function of the flagellar protein export apparatus are well conserved among bacterial species [5]. Interestingly, it has been reported that the *B. subtilis* export gate complex also serves as a platform for the assembly of a nanotube as a ubiquitous organelle involved in cell–cell exchange of proteins or plasmids when the export gate complex is not incorporated into the MS ring [33]. Thus, the *B. subtilis* flagellar protein export machinery has two distinct functions.

## 3 Ion specificity of the *B. subtilis* flagellar motor

**Fig. 4:**
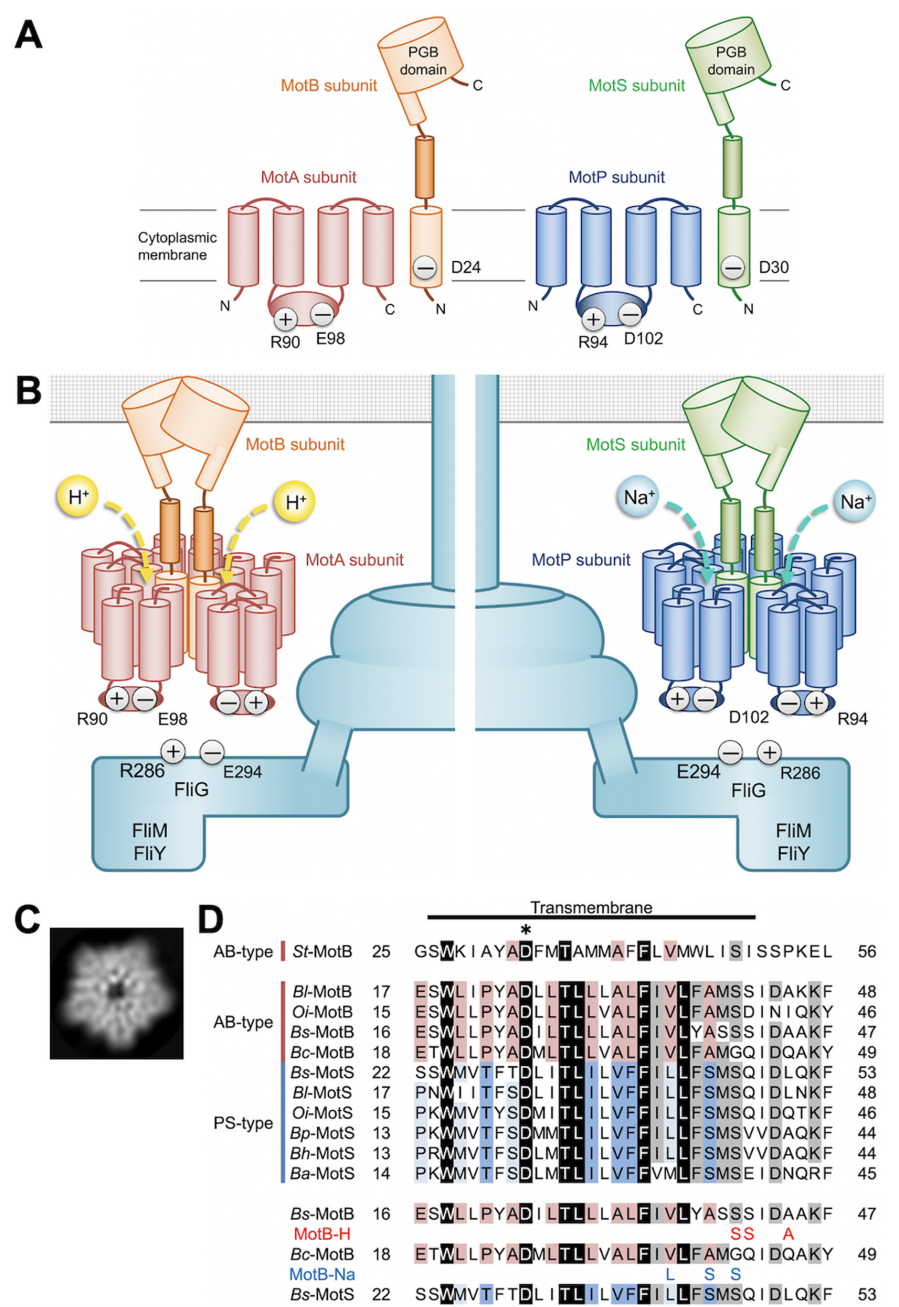
*B. subtilis* flagellar motor structure. (A) Topology model for MotA, MotB (left), MotP and MotS (right). MotA has four transmembrane helices and a large cytoplasmic domain between 2nd and 3rd of the transmembrane helices. Two highly conserved charged residues (Arg-90 and Glu-98 in MotA and Asp-102 and Arg-94 in MotP) are involved the interaction with the rotor protein FliG. MotB has a single transmembrane helix, a highly flexible linker and a large peptidoglycan binding (PGB) domain that anchors the stator complex to the peptidoglycan layer. A highly conserved Asp residue (Asp-24 in MotB and Asp-30 in MotS) is an ion-binding site in the ion channel. (B) Schematic diagram of the *B. subtilis* flagellar motor. MotA and MotB forms a H^+^ channel complex whereas MotP and MotS forms a Na^+^ channel. Recent cryoEM image analyses have shown that the MotAB complexes are composed of five copies of MotA and two copies of MotB. The protonation and deprotonation of a conserved Asp residue cause the conformational changes of a large cytoplasmic domain of MotA, allowing the rotor to spin in CCW or CW direction. The rotor is composed of three cytoplasmic proteins, namely FliG, FliM and FliY. FliG is directly involved in torque generation for flagellar rotation. Two highly conserved charged residues, Arg-286 and Glu-294, in *Bs-*FliG is responsible for electrostatic interactions with large cytoplasmic domains of MotA and MotP. (C) 2D-class average of the end-on view of purified MotPS complex using cryoEM image analysis. The MotPS complex has a 5-fold rotational symmetry in a way similar to the MotAB complex. (D) Multiple sequence alignment of *St*-MotB (UniProt ID: P55892), *Bl*-MotB and -MotS (*B. licheriformis*: Q65KJ0 and Q65G30), *Oi*-MotB and -MotS (*O. iheyensis*: Q8CX95 and Q8EP91), *Bs*-MotB and -MotS (P28612 and P39064), *Bc*-MotB (*B. clausii*: Q5WGI6), *Bp*-MotB (*B. peudofirmus*: D3FX74), *Bh*-MotB (*B. halodurans*: Q9K7W9), *Ba*-MotB (*B. alcalophilus*: G9I2I5) (upper panel). A highly conserved Asp residue involved in the ion binding was highlighted with an asterisk. Highly and relatively well conserved residues are shown by black and gray boxes, respectively. Red and blue boxes represent conserved residues in the MotB and MotS proteins, respectively. The G42S/Q43S/Q46A (red) and V37L/A40S/G42S (blue) triple mutations allow *Bc*-MotB to behave like the H^+^-type *Bs*-MotB and Na*^+^*-type MotS subunits, respectively, over a wide range of external pH (lower panel). (For interpretation of the references to color in this figure legend, the reader is referred to the web version of this article.)

The transmembrane ion channel stator complex is composed of three structural parts (Fig. 4A, B): a cytoplasmic domain interacting with the rotor protein FliG [34], [35], [36], a transmembrane ion channel domain involved in an inward-directed ion flow [37], [38] and a peptidoglycan-binding(PGB) domain that anchors the stator complex to the PG layer [39], [40]. A flexible linker connecting the ion channel domain with the PGB domain suppresses premature ion translocation through the ion channel before this ion channel complex becomes an active stator unit in the motor [41], [42]. This flexible linker also regulates the binding affinity of the PGB domain for the PG layer in a load-dependent manner [43]. The inward flow of ions through the ion channel causes a large conformational change in the cytoplasmic domain that allows the rotor to generate torque to rotate in CCW or CW direction (Fig. 4B) [44], [45].

The transmembrane helix (TMH) of MotB forms a H^+^ translocation pathway along with TMH-3 and TMH-4 of MotA (Fig. 4A). A universally conserved aspartic residue of MotB-TM (Asp-32 of *E. coli* MotB and Asp-33 of *Salmonella* MotB) plays an essential role in the H^+^ channel function [46], [47]. Two recent high-resolution cryoEM image analyses of the MotAB complex have shown that five copies of MotA and two copies of MotB form a transmembrane H^+^ channel complex with two distinct H^+^ pathways [48], [49]. Consistently, purified MotPS complex of *B. subtilis* exhibits a clear five-fold rotational symmetry in its cryoEM images (Fig. 4C) [Terahara, T., Kato, T., and Namba, K., unpublished data], suggesting that MotP and MotS form a Na^+^ channel complex with a similar structure to the MotAB complex.

Asp-24 of *Bs-*MotB and Asp-30 of *Bs-*MotS are H^+^ and Na^+^-binding sites, respectively (Fig. 4A, D) [50]. How do the MotAB and MotPS stator complexes select H^+^ and Na^+^ as the coupling ion, respectively? Extremely alkalophilic *Bacillus* species are known to possess only the *motPS* genes on their genome. In contrast, an alkalophilic bacterium, *B. clausii*, which can grow in highly alkaline environments, encodes only the H^+^-type *motAB* stator genes on the genome but not the *motPS* genes. The flagellar motor of *B. clausii* is powered by PMF at neutral pH and sodium motive force (SMF) at alkaline pH, suggesting that the *Bc-*MotAB stator unit of *B. clausii* conducts H^+^ at neutral pH and Na^+^ at alkaline pH [51]. Multiple sequence alignments of the TMHs of the MotB and MotS proteins of different *Bacillus* species have clearly shown that MotB-TMH and MotS-TMH are highly conserved, but the 42nd amino acid residue is glycine instead of serine in *Bc*-MotB (Fig. 4D). Because glycine provides a conformational flexibility of a TMH of membrane protein, Gly-42 may allow Na^+^ to move to the conserved Asp residue of *Bc-*MotB-TMH in a pH-dependent manner because the diameter of Na^+^ is larger than that of H^+^. In addition, in most MotB homologues derived from *Bacillus* species, amino acid residues corresponding to Gln-43 and Gln-46 of *Bc-*MotB are Ser/Asp and Ala/Ile, respectively (Fig. 4D), suggesting that these two Gln residues of *Bc-*MotB also contribute to pH-dependent Na^+^ flow through the ion channel of the *Bc-*MotAB complex. In fact, the introduction of the G42S/Q43S/Q46A triple mutation into *Bc*-MotB results in the loss of the Na^+^ channel activity of the *Bc*-MotAB stator complex [51]. Furthermore, amino acid residues of MotS corresponding to Val-37, Ala-40 and Gly-42 of *Bc-*MotB are Leu, Ser and Ser, respectively, and the V37L/A40S/G42S triple mutation actually allows the *Bc*-MotAB stator complex to conduct only Na^+^ over a wide range of external pH [51]. These observations lead to a plausible hypothesis that *Bc*-MotB-TMH has a pH-dependent ion filter and that Val-37, Ala-40, Gly-42, Gln-43 and Gln-46 of *Bc*-MotB contribute to the ion selectivity in an external pH-dependent manner. Thus, the periplasmic side of MotB-TMH and MotS-TMH seems to serve as an ion filter.

Although a valine residue in the H^+^-type MotB protein and a leucine residue in the Na^+^-type MotS protein serve as an important residue involved in the ion selectivity as stated above, the amino acid residue at this position of MotS (*Ba-*MotS) derived from an alkalophilic bacterium, *B. alcalophilus*, is methionine (Fig. 4D). The flagellar motor of *B. alcalophilus* is driven by SMF across the cell membrane. Interestingly, this flagellar motor also utilizes K^+^ as the coupling ion to drive flagellar motor rotation, suggesting that the *Ba-*MotPS stator complex conducts both Na^+^ and K^+^ to generate torque for high-speed motor rotation [52]. When this methionine residue is replaced by leucine, this mutant *Ba-*MotPS stator complex shows only the Na^+^ channel activity, and its K^+^ channel activity is completely lost, indicating that this methionine residue is critical for K^+^ recognition. Recently, it has been reported that not only MotS-TMH but also the MotP subunits contribute to efficient selection of K^+^ [53].

## 4 Torque-speed relationship of the *B. subtilis* flagellar motor

The *B. subtilis* BR151MA strain encodes two distinct types of stator units on its genome: H^+^-type MotAB and Na^+^-type MotPS. A deletion of the *motAB* genes causes a loss-of-motility phenotype even in the presence of the *motPS* genes. In contrast, disruption of the *motP* and *motS* genes has no impact on swimming motility, indicating that the MotAB stator complex is dominant for swimming motility of planktonic BR151MA cells under various experimental conditions [11]. In contrast, when the expression level of the MotPS complex is significantly increased by a mutation in a stem loop located between the *ccpA* and *motP* genes, MotPS-dependent motility is observed when external pH, external Na^+^ concentrations and fluid viscosity are high enough [12]. These observations raise interesting questions of why the over-expression of the MotPS complex is required for flagella-driven motility and why the assembly of the MotPS complex into the motor is dependent on external pH, the external Na^+^ concentration and the fluid viscosity.

**Fig. 5:**
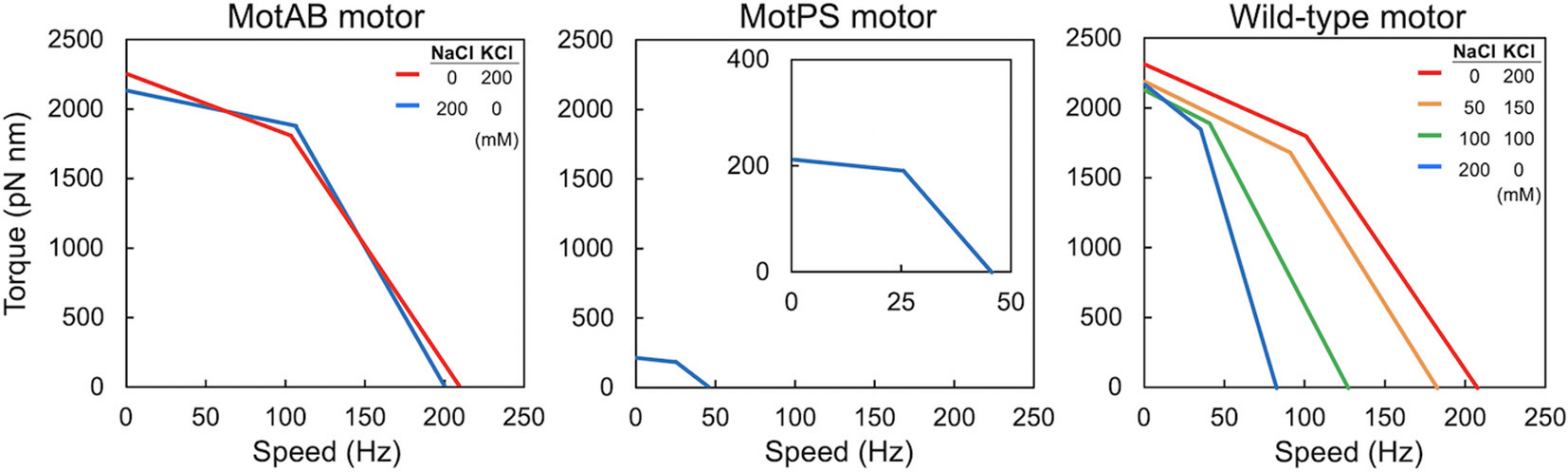
Torque-speed curve of the *B. subtilis* MotAB (left panel), MotPS (middle panel), wild-type motors (right). The NaCl concentrations in a buffer are 0 (red line), 50 (orange line), 100 (green line), 200 mM (blue line), respectively. The expanded curve of MotPS motor is shown in inset. These torque-speed curves were generated using datasets published in references 13 and 14. (For interpretation of the references to color in this figure legend, the reader is referred to the web version of this article.)

Precise measurements of motor rotation over a wide range of external load provide insights into the torque generation mechanism of the flagellar motor. In order to accurately measure the rotational speeds of the flagellar motor over a wide range of external load, the flagellar motor is labelled with a latex bead, and then the bead images are captured by a high-speed camera with high temporal and special resolutions [6]. Because a deletion of the D3 domain of *E. coli* and *Salmonella* FliC allows latex beads to be directly attached to the flagellar filament [54], [55], [56], residues 141-214 of the D1 domain of Hag was replaced by the D2 domain of *St-*FliC (residues 142–203 and 293–395) to efficiently label the *B. subtilis* flagellar filament with a bead (Fig. 2C and 3B) [14]. So far, the torque versus speed relationships of the flagellar motors of the wild-type (*motAB^+^ motPS^+^*), MotAB (*motAB^+^* Δ*motPS*) and MotPS (Δ*motAB motPS^+^*) strains have been investigated over a wide range of external load (Fig. 5) [13], [14].

The MotAB motor displays a typical torque-speed curve with a gradual decrease of torque in a high-load, low-speed regime and a rapid drop in a low-load, high-speed regime, as a function of speed (Fig. 5) [14], in a way similar to those of the *E. coli* and *Salmonella* flagellar motors [54], [55], [56]. The maximum torque at high load and maximum rotational speed at low load are estimated to be about 2000 pN nm and about 200 revolutions per second (rps), respectively, in the presence and absence of 200 mM Na^+^ (Fig. 5) [14]. In contrast, for the MotPS motor, which requires Na^+^ as the coupling ion, when MotP and MotS are expressed from the *P_motA_* promotor at the *amyE* locus in the genome, the maximum torque at high load and the maximum rotational speed at low load are estimated to be about 200 pN nm and about 50 rps, respectively, only in the presence of 200 mM Na^+^ (Fig. 5) [14]. A single MotPS stator unit produces nearly the same torque (ca. 200 pN nm) as that of a single MotAB stator unit [14]. Because the maximum torque produced by the flagellar motor is dependent on the number of active stator units in the motor [55], the number of active stator units in the MotAB and MotPS motors are estimated to be ten and one, respectively [14]. When the MotPS complex is expressed from the *P_motA_* promotor, the expression levels of MotP and MotS are increased by about 5-fold compared to those expressed from the *P_ccpA_* promoter [14]. Because a depletion of the MotAB complex abolishes flagella-driven motility even in the presence of the MotPS complex, these experimental results strongly suggest that the binding affinity of the MotPS complex for the motor is much weaker than that of the MotAB complex. Because the maximum rotation speed of the motor is limited by the rate of torque generation cycle of the motor [57], the rate of conformational changes of the MotPS stator coupled with the Na^+^ flow seems to be about 4-fold slower than that of the MotAB stator coupled with the H^+^ flow [14].

The maximum torque produced by the wild-type BR151MA motor is constant over a wide range of external Na^+^ concentration but the maximum speed decreases from ca. 200 to 80 rps with an increase in the external Na^+^ concentration from 0 to 200 mM (Fig. 5) [13]. In contrast, such a rotational speed reduction is not seen in the absence of the MotPS stator complex [14]. These observations suggest that the wild-type motor accommodates both H^+^-type MotAB and Na^+^-type MotPS stator units in the presence of NaCl, with more Na^+^-type MotPS stator units at higher Na^+^ concentration. Interestingly, MotPS-driven motility is seen in the absence of the MotAB complex only when the MotPS complex is over-expressed [12]. Because MotP and MotS are expressed at a relatively low level from the *P_ccpA_* promoter, there is the possibility that the MotPS complex may require pre-assembled MotAB stator complex to become an active Na^+^-coupled stator unit around the rotor.

## 5 Polysaccharide- and Na^+^-dependent stator assembly mechanism

**Fig. 6:**
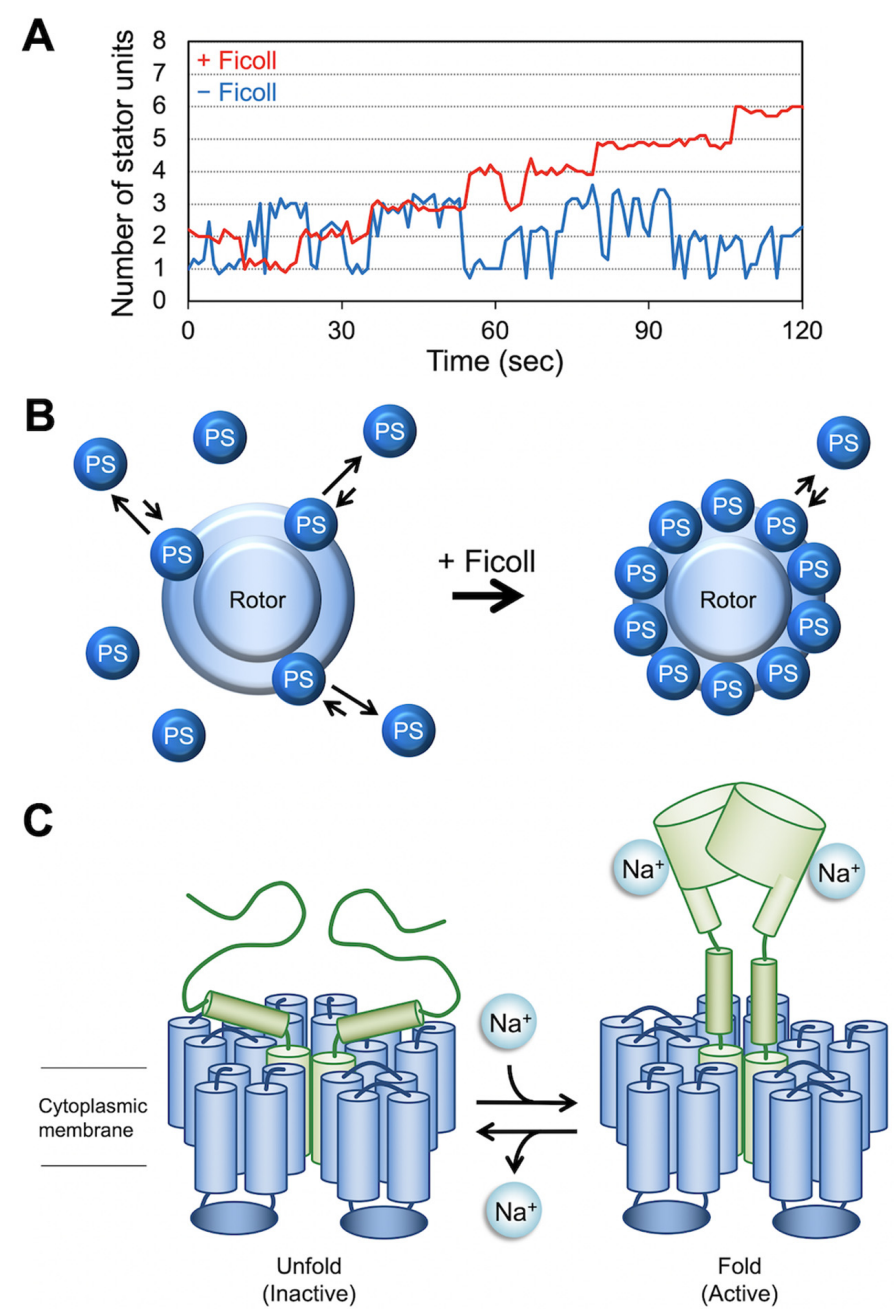
Viscosity and Na^+^-dependent MotPS assembly. (A) Rotation measurement of a single flagellar motor containing only the MotPS stator units in the presence and absence of 10% Ficoll 400. The MotPS complex was expressed from an IPTG-inducible P*_grac_* promoter by adding IPTG, and then rotational speeds were recorded by tracking the position of 1.0-μm beads attached to the partially sheared sticky filament. This trace was generated using datasets published in reference 13. (B) Ficoll-dependent assembly and disassembly mechanism of the MotPS complex. An increase in viscosity by adding Ficoll 400 does not affect the association rate of the MotPS complex, but considerably slows down the dissociation rate of the MotPS complex due to an increase in the binding affinity of the PGB domain of MotS to the PG layer, thereby increasing the number of active MotPS stator units around the rotor. (D) Na^+^-induced structural transition of the PGB domain of MotS. The binding of Na^+^ to the PGB domain of MotS induces a structural transition from an unfolded to a folded conformation, allowing the MotPS complex to become an active Na^+^-type stator unit in the motor.

**Fig. 7:**
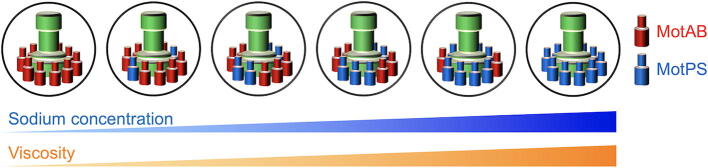
Autonomous stator exchange mechanism of the *B. subtilis* flagellar motor. The MotAB and MotPS stator units autonomously modulates their binding affinities for the motor in response to changes in external Na^+^ concentration and fluid viscosity. As a result, the number of active MotPS units in the motor increases with an increase in both extracellular Na^+^ and polysaccharide concentrations by efficient replacement of the MotAB stator complex by the MotPS complex to maintain the optimal motor performance under various environmental conditions.

Each stator unit assembles to and disassembles from the rotor during motor rotation in an external load-dependent manner, suggesting that the stator complex is not permanently fixed in a place around the rotor [58], [59], [60]. The MotAB motor shows a rather stable rotation speed, but the MotPS motor shows quite frequent accelerations and decelerations of rotation in the motility buffer solution at low fluid viscosity (Fig. 6A), indicating that the MotPS stator unit dissociates from and associate with the rotor more frequently than the MotAB stator unit (Fig. 6B) [14]. In contrast, an increase in viscosity by adding Ficoll 400, which is a neutral, highly branched, hydrophilic polysaccharide, suppresses such speed fluctuations (Fig. 6A) and increases the number of active MotPS stator units from one to ten in the motor of the MotPS strain (Fig. 6B) [14]. This suggests that the binding affinity of the MotPS stator unit for the motor increases by about 10-fold with a sufficient increase in fluid viscosity. Domain exchange experiments have shown that the PGB domain of the MotPS complex serves as a viscosity sensor to modulate the binding affinity of the PGB domain for the PG layer in a viscosity-dependent manner [14]. Recently, it has been shown that the number of active MotAB stator units in the motor of the MotAB strain decreases from ten to five with an increase in viscosity by adding Ficoll 400 [61], suggesting that the binding affinity of the MotAB stator unit for the motor decreases as the fluid viscosity increases, in an opposite manner to that of the MotPS complex. Furthermore, Ficoll 400 directly affects the binding affinities of the MotAB and MotPS stator complexes for the motor in a concentration-dependent manner even at the same load [14], [61], suggesting that both MotAB and MotPS stator complexes are also a polysaccharide sensor that detects changes in the extracellular polysaccharide concentration in the environment. Interestingly, the torque produced by the wild-type BM151MA motor remains constant over a wide range of the extracellular polysaccharide concentration [61], suggesting that the MotAB and MotPS stator units autonomously modulate their binding affinities for the motor in response to environmental changes to maintain the optimal motor performance under various environmental conditions (Fig. 7).

The wild-type BM151MA motor contains only the MotAB stator units around the rotor in the absence of Na^+^. In contrast, it becomes a hybrid engine containing both H^+^-type MotAB and H^+^-type MotPS stator units around the rotor when the external Na^+^ concentration is elevated [13]. How does the MotPS stator complex assemble to and dissociate form the motor in a Na^+^-dependent manner? High-speed atomic force microscopy observation of purified MotPS complex has revealed that the PGB domain of the MotPS complex adopts a folded and unfolded conformation in the presence and absence of 150 mM NaCl, respectively, and that the order-to-disorder structural transition of this PGB domain is reversible (Fig. 6C) [13]. Because Na^+^ directly stabilizes the folded conformation of the PGB domain of the MotPS stator complex [13], this suggests that the PGB domain of the MotPS stator complex serves as a Na^+^ sensor to detect changes in the external Na^+^ concentration to facilitate the assembly-disassembly cycle of the MotPS complex in a Na^+^-dependent manner.

## 6 Summary and perspective

The *B. subtilis* flagellar motor serves not only as a motility machine but also as a biosensor to sense solid surfaces to facilitate lifecycle transitions such as cell differentiation and biofilm formation [62], [63], [64]. The *B. subtilis* flagellar motor has adapted the structural dynamics and assembly of its stator units to function in various environments where *B. subtilis* cells live and survive. The *B. subtilis* flagellar motor is powered by two distinct types of stator units, H^+^-type MotAB and Na^+^-type MotPS. The MotAB complex is well known as a dominant stator unit in planktonic motile cells, but the number of functional MotPS stator units in the motor is increased from one to ten by elevated Na^+^ and polysaccharide concentrations (Fig. 7) [13], [14]. Interestingly, it has been shown that both MotAB and MotPS complexes not only detect changes in the fluid viscosity but also in the extracellular polysaccharide concentration [14], [61]. Thus, the MotAB and MotPS stator units autonomously modulates their binding affinities for the motor in response to environmental changes to maintain the optimal motor performance under various environmental conditions (Fig. 7). However, it still remains unknown why the rate of the mechanochemical coupling reaction cycle of the MotPS complex is 4-fold slower than that of the MotAB complex, how Na^+^ stabilizes the folded state of the PGB domain of the MotPS stator complex, and how the MotAB and MotPS complexes sense extracellular polysaccharides as well as fluid viscosity to modulate their binding affinity for the motor. We are currently clarifying these biologically important questions.

## CRediT authorship contribution statement

**Naoya Terahara:** Conceptualization, Writing - original draft, Funding acquisition. **Keiichi Namba:** Writing - review & editing, Project administration, Funding acquisition. **Tohru Minamino:** Writing - review & editing, Supervision, Funding acquisition.

## Declaration of Competing Interest

The authors declare that they have no known competing financial interests or personal relationships that could have appeared to influence the work reported in this paper.
